# Prospective correlation between the patient microbiome with response to and development of immune-mediated adverse effects to immunotherapy in lung cancer

**DOI:** 10.1186/s12885-021-08530-z

**Published:** 2021-07-13

**Authors:** Justin Chau, Meeta Yadav, Ben Liu, Muhammad Furqan, Qun Dai, Shailesh Shahi, Arnav Gupta, Keri Nace Mercer, Evan Eastman, Taher Abu Hejleh, Carlos Chan, George J. Weiner, Catherine Cherwin, Sonny T. M. Lee, Cuncong Zhong, Ashutosh Mangalam, Jun Zhang

**Affiliations:** 1 Division of Hematology, Oncology, and Blood & Marrow Transplantation, University of Iowa Hospitals and Clinics, Iowa City, USA; 2 Department of Pathology, University of Iowa Hospitals and Clinics, Iowa City, USA; 3 Department of Electrical Engineering and Computer Science, University of Kansas, Lawrence, USA; 4 Division of Medical Oncology, Department of Internal Medicine, University of Kansas Medical Center, Kansas City, USA; 5 Birla Institute of Technology and Science Pilani, KK Birla Goa Campus, Zuarinagar, India; 6 Holden Comprehensive Cancer Center, University of Iowa Hospitals and Clinics, Iowa City, USA; 7 Department of Surgery, University of Iowa Hospitals and Clinics, Iowa City, USA; 8 University of Iowa College of Nursing, Iowa City, USA; 9 Division of Biology, Kansas State University, Manhattan, USA; 10 Division of Hematology, Oncology, and Blood & Marrow Transplantation, Holden Comprehensive Cancer Center, University of Iowa Hospitals and Clinics, Iowa City, USA; 11 Department of Cancer Biology, University of Kansas Cancer Center, University of Kansas Medical Center, Kansas City, USA

**Keywords:** Immunotherapy, Immune checkpoint, Microbiome, Lung cancer, Adverse effects, Toxicity, Response

## Abstract

**Background:**

Though the gut microbiome has been associated with efficacy of immunotherapy (ICI) in certain cancers, similar findings have not been identified for microbiomes from other body sites and their correlation to treatment response and immune related adverse events (irAEs) in lung cancer (LC) patients receiving ICIs.

**Methods:**

We designed a prospective cohort study conducted from 2018 to 2020 at a single-center academic institution to assess for correlations between the microbiome in various body sites with treatment response and development of irAEs in LC patients treated with ICIs. Patients must have had measurable disease, ECOG 0–2, and good organ function to be included. Data was collected for analysis from January 2019 to October 2020. Patients with histopathologically confirmed, advanced/metastatic LC planned to undergo immunotherapy-based treatment were enrolled between September 2018 and June 2019. Nasal, buccal and gut microbiome samples were obtained prior to initiation of immunotherapy +/− chemotherapy, at development of adverse events (irAEs), and at improvement of irAEs to grade 1 or less.

**Results:**

Thirty-seven patients were enrolled, and 34 patients were evaluable for this report. 32 healthy controls (HC) from the same geographic region were included to compare baseline gut microbiota. Compared to HC, LC gut microbiota exhibited significantly lower α-diversity. The gut microbiome of patients who did not suffer irAEs were found to have relative enrichment of *Bifidobacterium* (*p* = 0.001) and *Desulfovibrio* (*p* = 0.0002). Responders to combined chemoimmunotherapy exhibited increased *Clostridiales* (*p* = 0.018) but reduced *Rikenellaceae* (*p* = 0.016). In responders to chemoimmunotherapy we also observed enrichment of *Finegoldia* in nasal microbiome, and increased *Megasphaera* but reduced *Actinobacillus* in buccal samples. Longitudinal samples exhibited a trend of α-diversity and certain microbial changes during the development and resolution of irAEs.

**Conclusions:**

This pilot study identifies significant differences in the gut microbiome between HC and LC patients, and their correlation to treatment response and irAEs in LC. In addition, it suggests potential predictive utility in nasal and buccal microbiomes, warranting further validation with a larger cohort and mechanistic dissection using preclinical models.

**Trial registration:**

ClinicalTrials.gov, NCT03688347. Retrospectively registered 09/28/2018.

**Supplementary Information:**

The online version contains supplementary material available at 10.1186/s12885-021-08530-z.

## Background

For carefully selected patients, the advent of immunotherapy opened promising avenues of treatment and expectations for improved survival. With it arose the challenges of managing its immune-related adverse effects (irAEs). Attention has shifted to increasing immunotherapy efficacy in addition to mitigating the development of irAEs [1].

Cancer propagation is largely a result of the body’s inability to destroy mutated cells bearing aberrant antigen signatures; the disease can be framed as dysregulation of patient immunity [2, 3]. The bacterial composition of the gut has been theorized to yield carcinomodulatory effects [4–6]. Early studies demonstrated that mice with bacteria-deplete gut microbiomes were less likely to develop cancer after compared to those with microbiome intact [7, 8]. Dysbiosis, similarly, has been linked to carcinogenesis [9]. Atopic pulmonary disorders have been linked to specific changes in the gut microbiome: this concept of “crosstalk” may also occur in extrapulmonary organ systems [5, 10–14]. Pivotally, in a recent study, genetically engineered mice were transplanted with bacteria provoking intrapulmonary IL-17 inflammatory changes and shown to facilitate the development of lung cancer [15].

The influence of the microbiome in chemotherapy and immunotherapy efficacy has been prolifically documented. Enteric manipulation of bacteria has been linked to altered treatment efficacy: antibiotic-treated mice exhibited blunted immune response to both immunotherapy and radiation therapy [16]. In mouse models, *Bacteroides* and *Bifidobacterium* have been implicated in enhancement of anti-CTLA-4 activation and response to anti-PD-L1 therapy [17–19]. In human patients, *Akkermansia* levels positively correlated with partial response or stable disease, and in mouse models, repletion of *Akkermansia* via oral gavage was able to repotentiate tumor response to CTLA-4 and PD-L1 therapy [20].

Less evidence exists to support the microbiome’s role in development of irAEs. *Firmicutes* enrichment has been associated with development of immune-related colitis following treatment with ipilimumab, whereas corresponding enrichment in *Bacteroidetes* was associated with fewer episodes of colitis [21]. Mice repleted with *B. fragilis* species were less likely to develop immune-related toxicities after exposure to anti-CTLA-4 inhibitors [18]. Taken together, these findings raise the question of a relationship between systemic and local microbiota in cancer – specifically, whether microbiome sites both local and distant could be correlated to pulmonary tumorigenesis, immunotherapy response or development of irAEs.

We designed a prospective study to address three specific questions. Our study compares microbiome composition between LC and HC residing in the same geographic area. Second, we will evaluate for longitudinal correlations in the microbiome of three separate body sites – gut, buccal, and nasal, the latter two chosen for their proximity to and possible predictive surrogacy for the respiratory tract. This surrogacy is of particular interest in lung cancer: though the microbiome in distant body sites were previously found not to correlate with immunotherapy response in melanoma patients [22], it remains paramount to ascertain whether the respiratory tract microenvironment correlates to immunotherapy response and toxicity for lung cancer, especially considering aforementioned evidence that respiratory tract bacteria can facilitate lung cancer progression [15]. Lastly, we will analyze for associations between the microbiome and tumor response to immunotherapy and/or development of irAEs.

## Methods

### Patients

Patients 18 years or older with histopathologically confirmed LC whose treatment regimens included immunotherapy, either as monotherapy or in combination with chemotherapy, were eligible for this study. Exclusion criteria included active pregnancy, active recreational drug or alcohol abuse, and localized disease that could be managed definitively with surgery.

### Study design and treatments

**Fig. 1:**
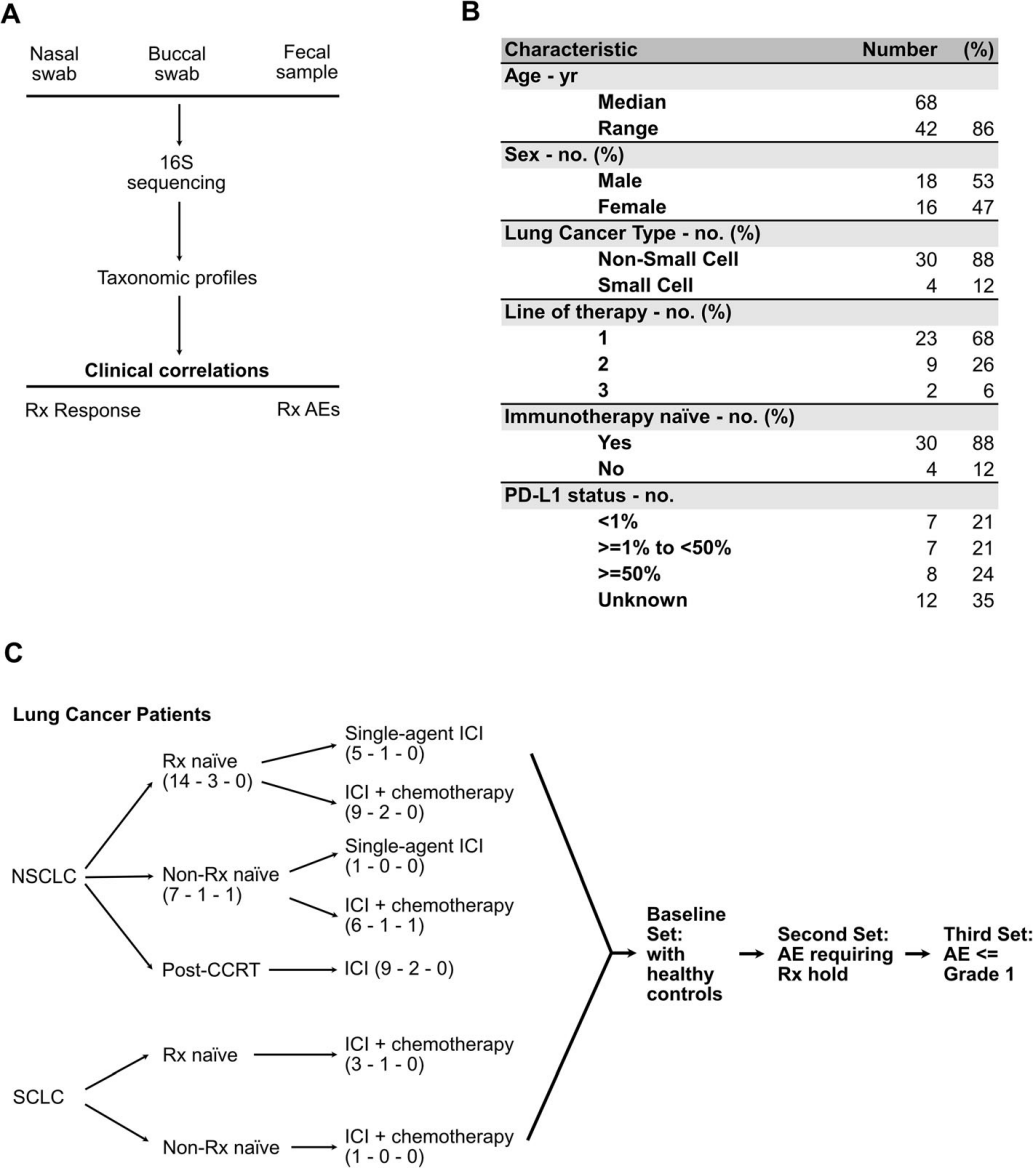
Description of patient cohorts and study schema. **(a)** Study schema showing the collection of microbiome samples from three separate body sites. Samples undergo 16S rRNA amplicon sequencing followed by taxonomic profiling. The resulting data is correlated to clinical outcomes such as response to ICI therapy or development of AEs. **(b)** An abbreviated demographics chart summarizing notable disease and patient characteristics of LC contributors. For granular individual characteristics, please refer to Supplemental Table 1. **(c)** Breakdown of patients belonging to each study cohort. ICI = immune checkpoint inhibitor. Checkpoint inhibitor status (number of patients enrolled – number of fecal samples that were unable to be analyzed or not submitted – number of nasal/buccal samples that were unable to be analyzed or not submitted

This prospective, single-center cohort pilot study was conducted at the University of Iowa Holden Comprehensive Cancer Center (HCCC) in Iowa, United States. The study was approved by the University of Iowa Hospitals and Clinics Institutional Review Board. Eligible patients visiting HCCC for LC treatment were screened, provided written informed consent, and enrolled in the study immediately prior to beginning immunotherapy or chemo-immunotherapy (Fig. 1a). Patients were enrolled between September 2018 and June 2019.

Patients were treated per guidelines for their disease subtype, overseen by participating oncologists whose subspecialty covered the patient’s primary diagnosis. Microbiome sample collection was triggered by the following events: (1) Prior to initiation of immunotherapy, (2) at the time immunotherapy was held due to concern for AE/irAE development, and (3) at improvement of AE/irAE severity to Grade 1 or less (Fig. 1c).

### Assessments

#### Microbiome sampling, DNA extraction, sequencing and analyses

Microbiome control comparisons were obtained from 32 previously identified healthy patient samples stored in a separate repository for a prior study (Cherwin et al., *Healthy Control for Microbiome, Cytokine, and Immunity Biomarker Analysis,* IRB-01, The University of Iowa, #201902825).

At enrollment, nasal and buccal mucosal swabs, and fecal samples were obtained. If a fecal sample could not be obtained at enrollment, the patient received a stool collection kit to return the sample prior to beginning immunotherapy.

Fecal samples were collected using a Commode Specimen Collection System, oral samples were collected using saliva collection tubes (Access-genetics, Eden Prairie, MN) and nasal samples were collected using ESwab™ (Copan Diagnostic, Murrieta, CA). All samples were then stored at − 80 °C prior to processing. DNA was extracted from the fecal, oral and nasal samples using DNeasy PowerLyzer PowerSoil Kit (Qiagen, Germantown, MD) 16S rRNA regions (V3–4) were amplified as previously described [23]. DNA library for sequencing was prepared for Illumina MiSeq; we used the Nextera XT Index kit to attach dual index adapters. Each library was prepared by diluting the samples to 5 ng/μL and equal volumes were mixed to 4 nM. We quantified the DNA concentration by Qubit (Thermo Fisher Scientific, USA). We carried out the library preparations according to the 16S library preparation protocol of Illumina (Illumina, San Diego, Cam USA), and sequenced the libraries using the MiSeq Reagent Kit v3 (600 cycles) for 300-bp pair-ends.

Raw reads were quality-controlled, merged, and mapped to the 16S reference database (SILVA 13.2 [24]) using DADA2 [25] to generate OTU clusters. When running DADA2, the accepted amplicon lengths were set to between 240 and 260 bp (with parameter “truncLen”), followed by trimming the leading 15 bp low-quality region (with parameter “trimLeft”). Pair-end reads remaining after each stage of DADA2 are included in **QC and Read Statistics.** The average percent of remaining input reads (non-chimera) ranged from 68% in the nasal response cohort to 93% in the buccal toxicity cohort. The resulting OTU clusters were then analyzed using MicrobiomeAnalyst to compute α-diversity, β-diversity and assess differential abundance. When running MicrobiomeAnalyst [26], OTU count were first transformed into centered log ratio (CLR); taxa with little variance between conditions (the lower 0.5% quantile) were not considered because they are less informative in comparative studies (by setting the inter-quantile range to 0.5%). METAGENassist [27] was used to perform partial least squares-discriminant analysis (PLS-DA). Excepting adjustments mentioned above, default parameters for all programs were used across the entire study.

Fecal microbiome sequencing and analysis were initially conducted at the Iowa Institute of Human Genetics and confirmed separately at the University of Kansas. Nasal and buccal microbiome analysis was conducted at the University of Kansas.

#### Toxicity assessment

Toxicities/AEs were evaluated at time of patient presentation to clinic or in the event of acute hospitalization, documented by the treating oncologist in accordance with the Common Terminology Criteria for Adverse Events (CTCAE) Version 5.0. irAEs were defined as AEs consistent with an immune-mediated mechanism of action and requiring management with steroids or other immunosuppressants, and/or endocrine-targeted therapy for endocrinopathies [28, 29].

#### Response assessment

Patients were evaluated for response or progression via CT or PET/CT imaging. Imaging was obtained after 3 cycles’ therapy if the patient was treated with a single-agent regimen, and after 2 cycles with a doublet regimen. Responders were classified as patients who experienced complete response (CR) or partial response (PR) with a duration of at least 3 ~ 6 months after starting immunotherapy; those with stable or progressive disease were considered non-responders.

## Results

### Patient demographics

**Fig. 2:**
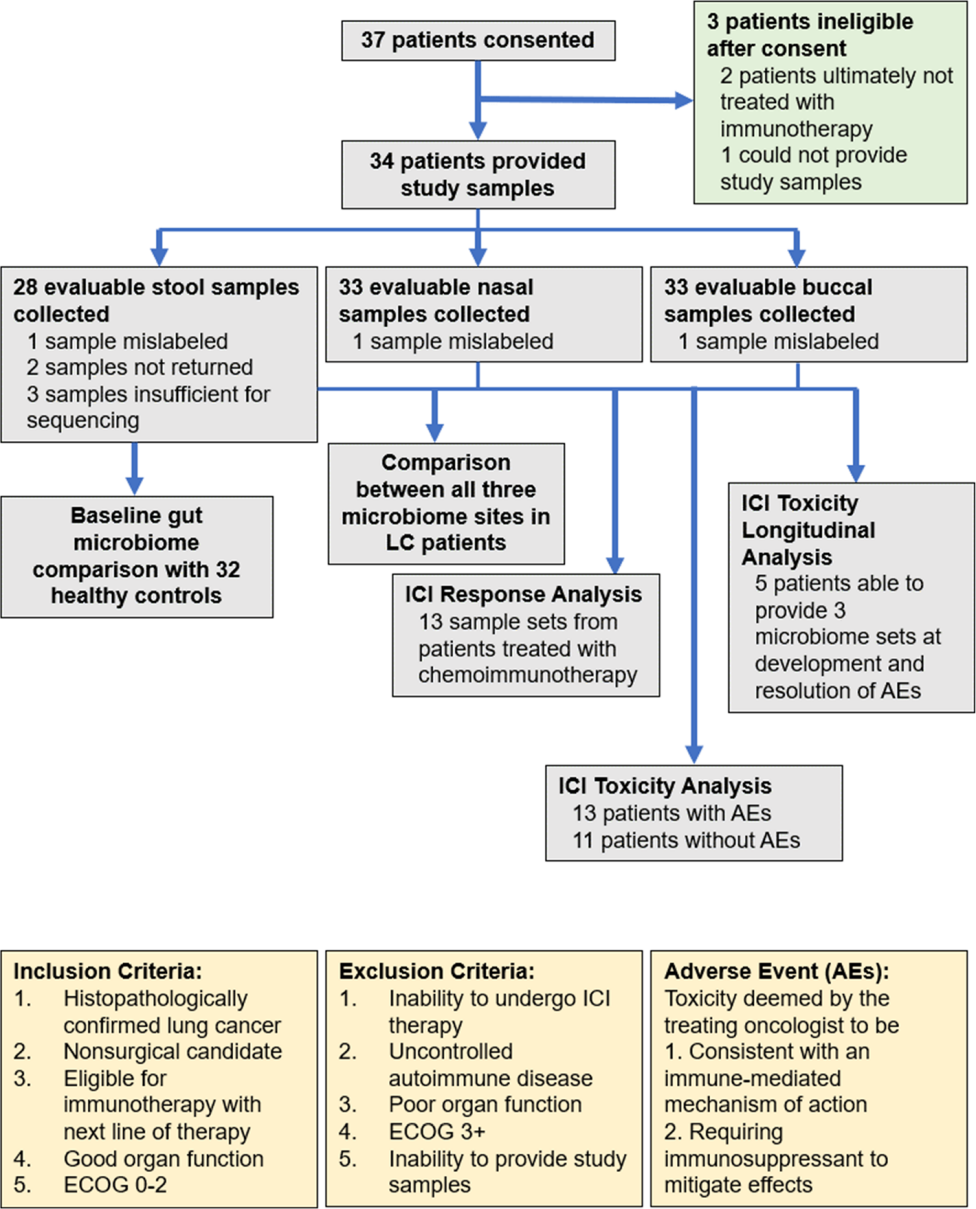
Flowchart of patients enrolled in the study. Breakdown of number of patients enrolled, were deemed ineligible for the study, as well as number of samples provided for each stage of analysis

37 patients were consented between September 2018 and June 2019. Patient and disease characteristics are summarized in Fig. 1b**,** with individual disease characteristics listed in **Supplemental Table** **1**, and three withdrawals prior to initiation of treatment outlined in **Supplemental Table** **3** as well as Fig. 2. Data collection and analysis was locked as of October 05, 2020; median follow-up was 12.2 months (range 0.33–24.3 months). Median progression-free survival was 4.1 months (range 1.4–12.2 months). Median overall survival was 10.0 months (range 0.3–19.8 months).

### Differences in gut microbiota composition between healthy controls and lung cancer patients

**Fig. 3:**
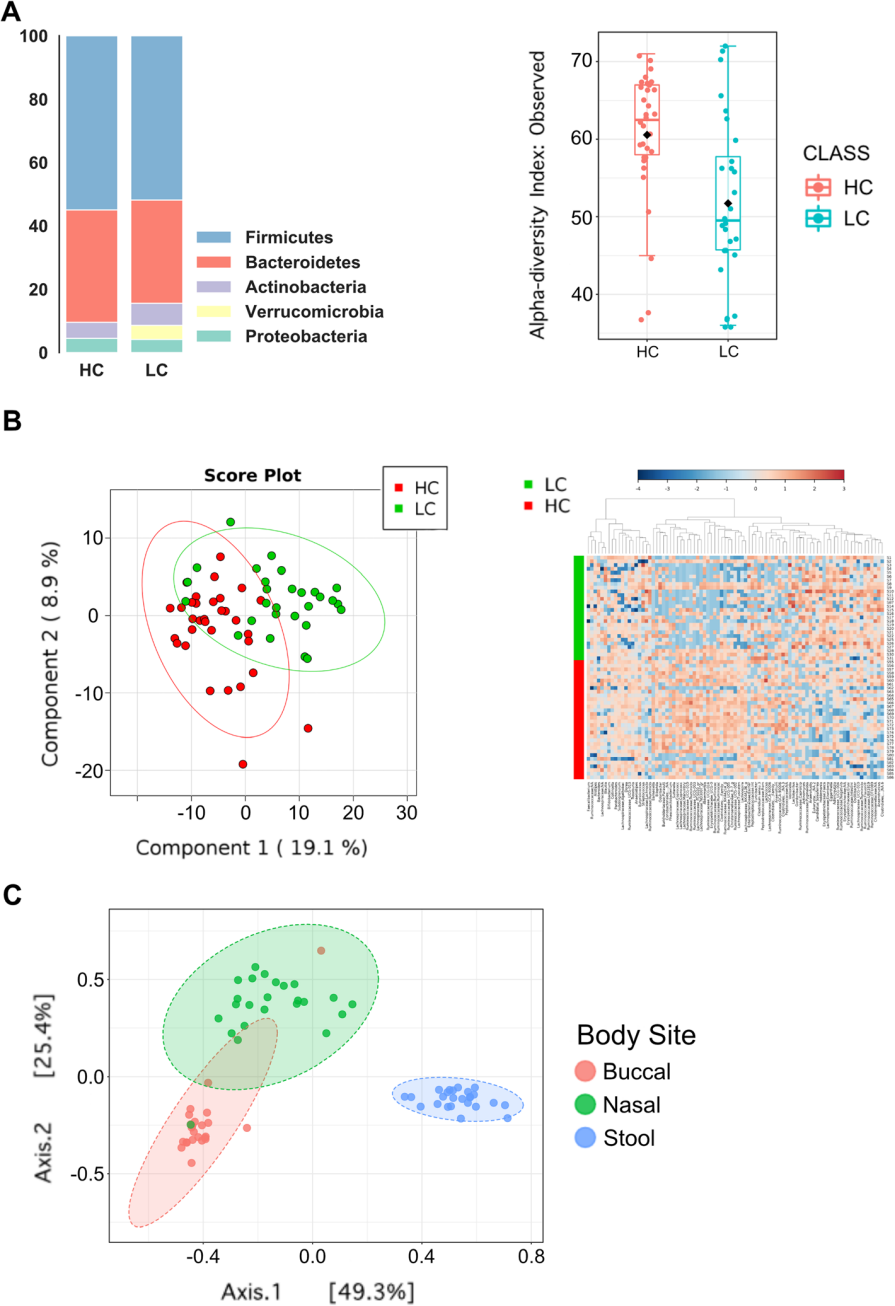
Baseline microbiome composition. Bar and heatmap plots comparing baseline gut, nasal and buccal microbiomes in LC compared to HC. **(a)** *Left:* Bar graph showing relative ratios of phyla constitution in LC and HC samples. *Right:* Box plot showing a statistically significant decrease in α-diversity when comparing LC to HC patients (*p* = 9.36 × 10^− 04^). **(b)** *Left:* 2-dimensional PLS-DA graph identifying notable differences in genus expression when comparing LC vs HC samples. *Right*: Heatmap showing genus level expression in LC patients (upper half) compared with HC (lower half). There is a notable difference at the genus level. **(c)** Principal coordinate analysis (PCoA) comparing the beta-diversity of buccal, nasal and gut microbiome in LC patients

Comparisons between LC and HC were restricted to fecal analysis as HC patients did not submit nasal or buccal samples. At the phylum level, LC samples were observed to exhibit lower relative abundances of *Firmicutes* and *Bacteroidetes*, but higher relative abundance of *Actinobacteria* and *Verrucomicrobia*. α-diversity (observed OTU) was found to be significantly lower in LC compared to HC samples (*p* = 9.36 × 10^− 04^) (Fig. 3a). At the genus level, notable differences were identified between HC and LC patients via PLS-DA as well as heatmap clustering (Fig. 3b). In univariate analysis, a number of taxa showed enrichment or depletion in the LC group compared to HC group: as an example, LC patients exhibited enriched baseline relative abundances of *Eggerthella* (*p* = 6.78 × 10^− 07^, FDR = 2.41 × 10^− 05^) and decreased *Lachnospira* (*p* = 2.00 × 10^− 04^, FDR = 1.58 × 10^− 03^). Please see **Supplemental Table** **4** for full list of bacteria with *p* and false discovery rate (FDR) values**.** As expected, β-diversity analysis using Bray-Curtis indices showed the gut microbiome was distinct from nasal and buccal samples **(**Fig. 3c**)**.

### Response to immunotherapy

Due to limited sample size, only patients receiving combined chemo-immunotherapy were used to determine the effect of microbiome on ICI response. Baseline nasal, buccal and fecal samples from 13 evaluable patients were included for analysis. Despite a consistent trend of higher baseline α-diversity among responders across all microbiome sites (**Supplemental Figure** **1****),** no statistically significant difference was observed.

**Fig. 4:**
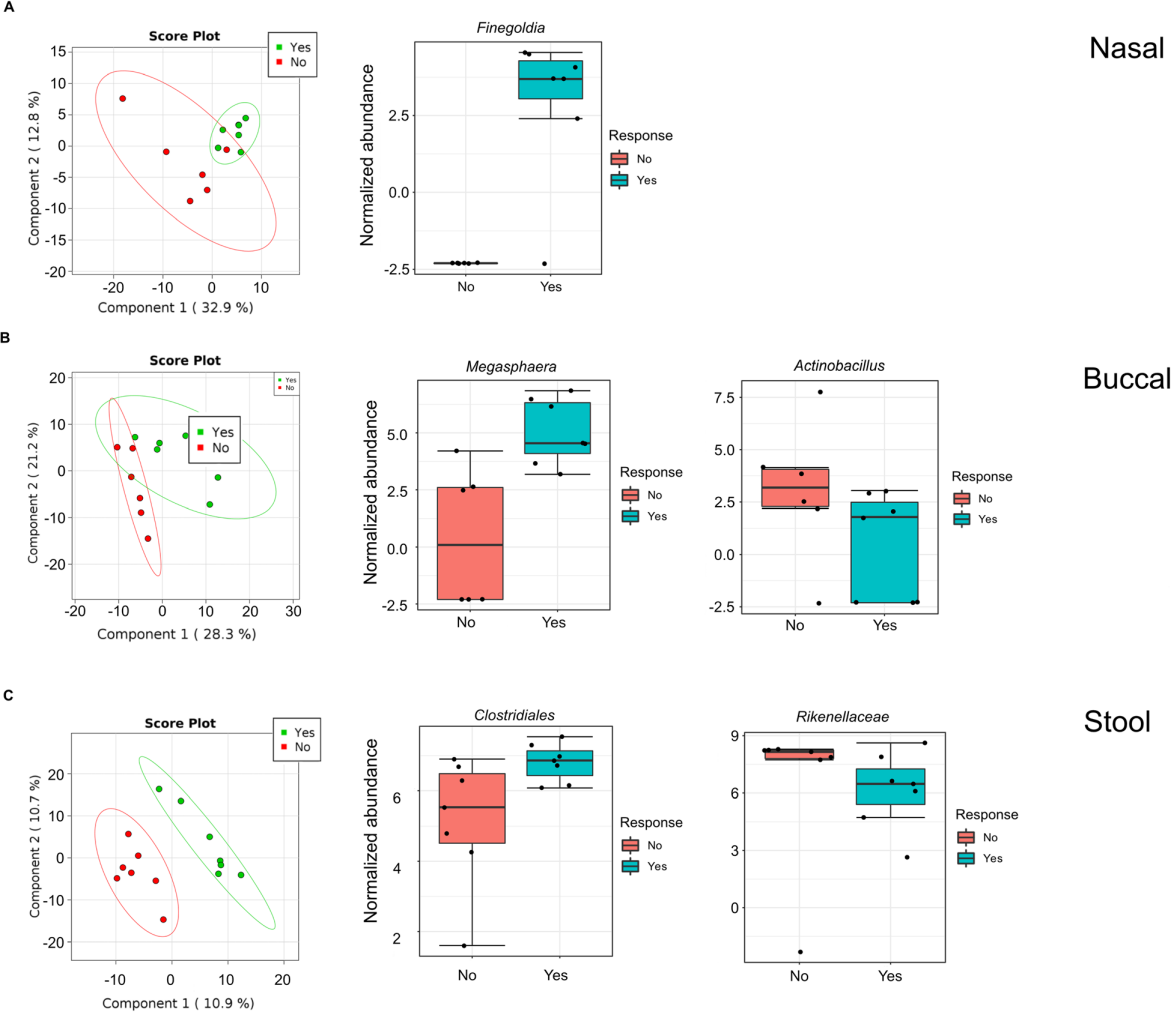
Response to immunotherapy. Microbiome changes notable in responders to ICI. Normalized data is presented in log-adjusted relative abundances. Left panels show PLS-DA graphs from nasal, buccal and gut sites all showing a microbiome separation when comparing ICI responders vs nonresponders. **(a)** Taxonomic profiling of nasal samples identified notable enrichment in *Finegoldia*, of phylum *Firmicutes*, in responders to ICI (*p* = 0.0005). **(b)** Buccal analysis of ICI responders show enrichment in *Megasphaera* of phylum *Firmicutes* (*p* = 8.6 × 10^− 03^) and decrease in *Actinobacillus* of phylum *Proteobacteria* (*p* = 9.7 × 10^− 03^). **(c)** In the fecal samples of ICI responders, *Clostridiales* was enriched (phylum *Firmicutes*, *p* = 0.017875) and *Rikenellaceae* decreased (phylum *Bacteroidetes, p* = 0.016013)

Nasal microbiome analysis identified statistically significant higher relative abundance of *Finegoldia* (of phylum *Firmicutes*), *p* and FDR < 0.05 (*p* = 5.21 × 10^− 04^, FDR = 0.018), in patients who enjoyed clinical response to chemoimmunotherapy (Fig. 4a). *Anaerococcus*, another bacteria of phylum *Firmicutes*, was significantly higher in responders (*p* = 2.93 × 10^− 04^, data not shown). In buccal samples, relative abundance of *Megasphaera* (phylum *Firmicutes*) was higher in responders (*p* = 8.6 × 10^− 03^), while *Actinobacillus* (phylum *Proteobacteria*) was lower (*p* = 9.7 × 10^− 03^) (Fig. 4b). In fecal samples, relative abundance of *Clostridiales* (phylum *Firmicutes*) was higher in responders (*p* = 0.017875) whereas *Rikenellaceae* (phylum *Bacteroidetes*) was lower (*p* = 0.016013) (Fig. 4c). In all three microbiome sites, *Firmicutes* bacteria were enriched in responders.

### Adverse effects

**Fig. 5:**
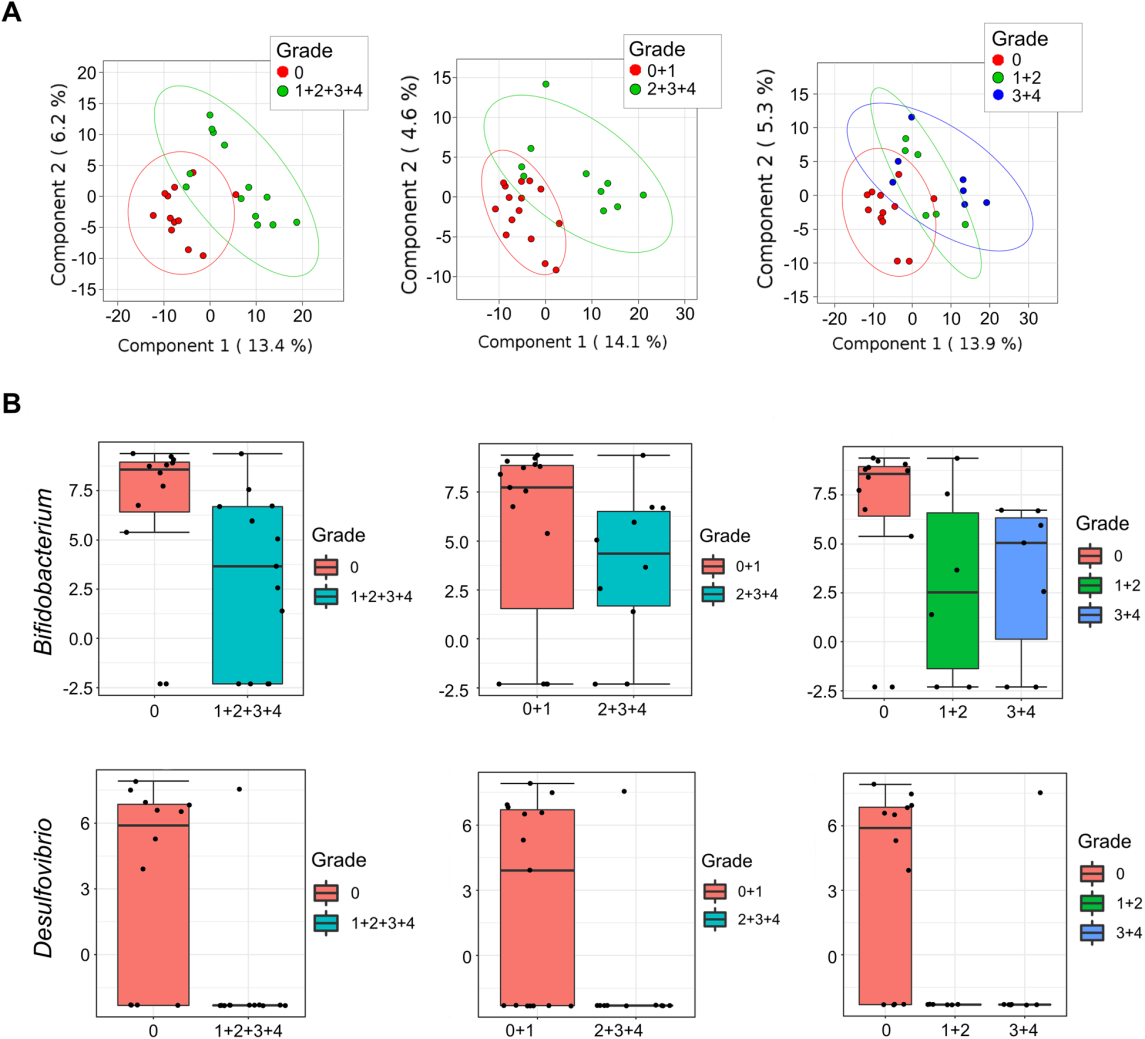
Toxicity analysis. (**a**) PLS-DA analysis showing significant microbiome differences between LC patients who experienced toxicities and those who did not using different grouping methods of irAE severities, e.g. grade 0 vs. grade 1 + 2 + 3 + 4; grade 0 + 1 vs. grade 2 + 3 + 4; and grade 0 vs. 1 + 2 vs. 3 + 4. **(b)** Normalized abundances of *Bifidobacterium* (phylum *Actinobacteria*) and *Desulfovibrio* (phylum *Proteobacteria*) showed enrichment in both bacteria in patients who developed less irAEs. All differences were statistically significant irrespective of categorization of AE severity

13 patients who experienced treatment related adverse effects provided fecal, buccal and nasal samples for analysis of irAEs (**Supplemental Table** **2**), along with 11 patients who did not develop irAEs. Taxonomic analysis identified multiple differences between microbiota of LC patients with and without adverse effects. At baseline, patients who experienced irAEs had a distinctly different fecal microbiome makeup compared to LC patients with fewer toxicities. Using stringent criteria, requiring *p* and FDR < 0.05 regardless of different grouping methods of AE severity, enrichment of *Bifidobacterium* (phylum *Actinobacteria*) and *Desulfovibrio* (phylum *Proteobacteria*) were significantly associated with lower incidence of irAEs. This observation was consistent irrespective of irAE severity grouping - i.e., CTCAE grade 0 vs 1 + 2 + 3 + 4 (*Desulfovibrio p* = 0.0002, *Bifidobacterium p* = 0.001), grade 0 + 1 vs grade 2 + 3 + 4 (*Desulfovibrio p* = 0.0077, *Bifidobacterium p* = 0.0004), or 0 vs 1 + 2 vs 3 + 4 (*Desulfovibrio p* = 0.0006, *Bifidobacterium* *p* = 0.001) **(**Fig. 5**)**. Under the same stringent criteria, buccal and nasal samples did not identify clear associations between the microbiome and irAEs, though differences in composition were observed using various grouping methods (**Supplemental Figure** **2**). Increasing the sample size by including patients who provided only nasal and buccal but not stool specimens also did not identify additional significant bacteria, irrespective of grouping (**Supplemental Figure** **3**).

### Longitudinal changes in microbiome in relation to toxicity

For longitudinal toxicity analysis, 11 patients submitted 2 sets of fecal samples; 3 patients submitted 3 sets. 13 patients submitted 2 sets of nasal and buccal samples; 5 patients submitted 3 sets of nasal and buccal samples. See **Supplemental Table** **2** for individual reasons for harvest of subsequent sample sets.

**Fig. 6:**
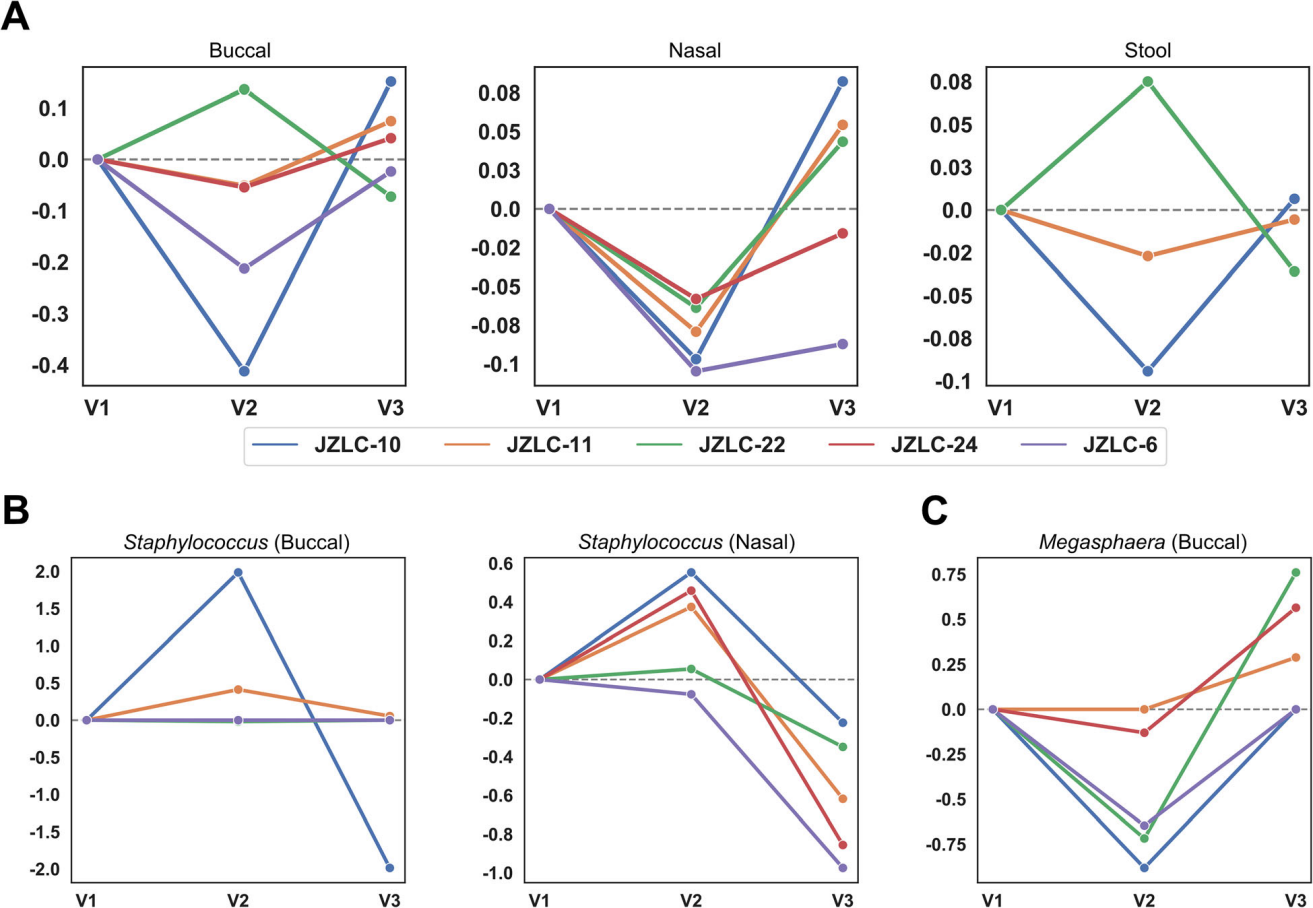
Longitudinal changes in microbiome with development and resolution of toxicities. **(a)** Analysis identified five patients who had submitted multiple samples during development and resolution of irAE. JZLC-24 and JZLC-6 did not have stool samples available for analysis but did submit all three sets of nasal and buccal samples. On the x-axis, sample collections are listed: V1, prior to initiation of immunotherapy; V2; at onset of toxicity, and V3, at resolution of irAE. The y-axis denotes the logarithmic (base 10) relative change in α -diversity compared to the previous visit, trended by the line plots, overlaid. Across nearly all sets of microbiome samples, a drop in microbiome α-diversity is observed at onset of irAE. At resolution of irAE to grade 1 severity or better, a third set of samples exhibit a trend toward either slowed rate of decrease in α-diversity or a reversal altogether toward baseline. **(b)** A consistent trend in increase of *Staphylococcus* at onset of toxicity and decrease with resolution of toxicity was also observed in the nasal samples (left), with a similar trend in buccal samples (right). **(c)** *Megasphaera*, a bacterium belonging to *Firmicutes*, was previously identified as being enriched in responders to immunotherapy. Here, it is also shown to decrease in buccal samples of patients who developed irAEs, then increasing in abundance with resolution of toxicity

For the 8 and 10 patients who submitted two sets of stool and nasal/buccal swabs for irAEs respectively, we found no statistically different changes in α-diversity, though a trend toward reduction in α-diversity in the gut microbiome at onset of toxicity was observed (**Supplemental Figure** **4**). In the 5 patients who provided all 3 sets of microbiomes, a consistent trend toward either slowed or reversed decrease in α-diversity with resolution of irAEs was observed (Fig. 6a), though complete recovery was not exhibited in this cohort.

Individual notable changes in the nasal and buccal microbiomes are included in Fig. 6b. Though none reached statistical significance, consistent increases in the relative abundance of *Staphylococcus* in nasal microbiome at onset of toxicities and concomitant decreases with resolution of toxicities were detected. In addition, the relative abundance of *Megasphaera* decreased in buccal samples with onset of irAE and increase with resolution of toxicities (Fig. 6c**)**.

## Discussion

In our cohort, LC patients were observed to have at baseline significantly decreased α-diversity in the gut microbiome. Decreased α-diversity is suggestive of a certain degree of dysbiosis, reminiscent of previous studies linking gut dysbiosis to carcinogenesis partly via leakage of microbial products that negatively affect the immune system [30]. Decreased α-diversity has been previously directly correlated with an active disease state [14, 31]. We had reported in earlier analyses an inversion in the ratio correlating *Firmicutes* and *Bacteroidetes* when comparing LC to HC samples [32]. This inversion was not upheld with increased sample size: when we presented our interim findings in 2019, we had accrued 9 LC patients to compare to 32 HC samples [33]. However, our sample size for comparison in this publication includes 28 LC patients, and our findings are concordant with another study that identified a relative decrease in *Firmicutes* abundance with simultaneous increased enrichment of *Bacteroidetes* in LC patients [34]. This could be due to improved elimination of confounders with increased sample size. As seen in **Supplemental Table** **1**, we performed a medicine reconciliation with patients prior to undergoing immunotherapy +/− chemotherapy and identified which of them had been taking antibiotics, probiotics or proton pump inhibitor therapies, as these have been shown to at least temporarily influence microbiome composition. Taking our small sample size into account, we were unable to identify any statistically meaningful signals attributing these variables to our study outcomes. As a medication reconciliation was a standard component of our enrollment process, we will continue to evaluate the potential value of these variables with future trials.

Multiple bacteria have been associated with potentiation of immunotherapy, including *Akkermansia, Faecalibacterium,* and *Bifidobacterium* [35]. Prior studies established that increased diversity in the microbiome is associated with response to immunotherapy in melanoma patients [22, 36, 37]. This correlation appears to have an amplifying effect on treatment efficacy: in a 2019 study, survival also seemed to increase with increasing α-diversity [37]. Though our study could not confirm those findings, likely due to underpowered sample size, this trend was observed.

Buccal and nasal samples yielded an increase in relative abundance of *Megasphaera* and *Finegoldia*, respectively, of phylum *Firmicutes*, in responders to combined chemoimmunotherapy; and increased *Actinobacillus*, a *Proteobacterium*, in nonresponders. In all three collection sites, *Firmicutes* bacteria (*Finegoldia* in nasal*, Megasphaera* in buccal*,* and *Clostridiales* in gut) were enriched in responders. Our recent systematic review of clinical studies suggests enrichment of *Firmicutes* in the gut microbiome correlates significantly with increasing chemoimmunotherapy response across various solid tumors [38]. *Firmicutes* bacteria are prominent producers of short-chain fatty acids (SCFAs), which have been linked to major immunoregulatory effects in the gut [30]. Preclinical studies have demonstrated the potential contribution of various SCFAs in patient immunity, particularly butyrate, which has been theorized at high concentrations to foster anti-tumor effects via activation of effector CD4 and CD8 responses [39–41]. The implications of a nasal or buccal sample being able to correlate with clinical outcomes in concordance with fecal samples would lend credence to the concept of immune systemic crosstalk, as well as improve the feasibility of utilizing the microbiome for predictive purposes. In our study, patients had a far easier time providing nasal and buccal swabs than fecal samples – compliance rate of nasal/buccal sample return was 97% - only one sample had been excluded due to mislabeling.

The significance of reduced relative abundance of *Actinobacillus* (phylum *Proteobacteria*) in the gut microbiome of chemoimmunotherapy responders is less clear. Increased *Proteobacteria* has been previously associated with dysbiosis [42, 43], and enterocolic inflammation possibly through local induction of intestinal Th17 cell responses [44]. *Helicobacter pylori*, a prominent *Proteobacterium*, has been found to utilize multiple mechanisms to incite local inflammation while simultaneously evading its own destruction via interleukin-33, which decreases interferon-γ production [45–47]. A similar mechanism may exist for *Actinobacillus*.

Our study made a novel association between decreased relative abundance of *Rikenellaceae* (phylum *Bacteroidetes*) and chemoimmunotherapy response. Current understanding of *Bacteroidetes’* function in response to ICI is mixed; *Bacteroidetes* have been demonstrated to increase regulatory T-cell differentiation as well as increase levels of IL-10 via use of Polysaccharide A, leading to upregulation of CTLA-4 expression [21]. It is important to note that certain species of *Bacteroidetes*, such as *B. thetaiotomicron,* and *B. fragilis* have been paradoxically shown to improve tumor control with CTLA-4 therapy [18]. ICI response with these specific species was theorized to be due to release of outer membrane vesicles containing enzymes that degrade gut mucin and improve presentation to dendritic cells, thus stimulating a stronger immune response [48]. It is possible this ability to potentiate immunotherapy is species-specific, not phylum-specific – this is indeed supported by findings from multiple studies across various solid tumors [49], and that the overall function of *Bacteroidetes*, specifically *Rikenellaceae*, could be immunosuppressive.

Our study is likely among the first to directly associate specific bacterial genera with irAE development. Particularly notable is association of *Bifidobacterium* enrichment with decreased irAEs. *Bifidobacterium* is a well-characterized probiotic and has been previously associated with alleviation of immunologic colitis caused by anti-CTLA-4 therapy: this effect is hypothesized to involve modulation of existing regulatory T-cells without major change on influx or distribution [50]. *Bifidobacterium* was found to reduce pro-inflammatory cytokines such as IL-17 and TNF, both of which play a critical role in development of irAEs [5, 49, 51, 52]. Interestingly, we also found that *Desulfovibrio* (phylum *Proteobacteria*) enrichment was significantly associated with decreased irAEs. Existing literature regarding its immunomodulatory behavior is scarce, and functional intraspecies differences have been identified [53]. Between strains, the rate of sulfate metabolism from SCFAs and thus possible consequent generation of enterotoxic hydrogen sulfides can vary dramatically [54, 55]. Whether the strain we identified in our study specifically dampens inflammatory response to ICI will require more focused analysis. In addition to development of lung cancer and poor response to ICI, *Proteobacteria* has been correlated with lower incidence of irAEs [56, 57]. It is unclear why our gut microbiome toxicity findings were not mirrored in nasal or buccal sets as they were with response data. We speculate irAEs are primarily a systemic response to which the gut microbiome wields significant influence given a much larger commensal bacteria mass, whereas ICI response is primarily determined by the tumor immune microenvironment and hence is likely modulated by local (e.g. nasal and buccal) microbiota as well.

Longitudinally collected samples appeared to show consistent loss of α-diversity in buccal, nasal and gut microbiomes with onset of irAEs, with a trend toward either decreased rate of diversity loss or outright improvement at resolution of toxicity. The lack of statistical significance here may simply be a matter of sample size. It is possible that patients who experience irAEs eventually recover baseline microbiome composition with longer follow-up: future studies designed to obtain samples far after resolution may confirm recovery of baseline microbiome diversity. It also remains unclear whether increased biodiversity in HCs is reflective of bio-reductive activity by malignancy or a baseline trait of the host in general, which could also reflect better overall health and performance status and thus prolong survival [58].

We identified two specific phenomena with longitudinal sampling. First, we noted an increase in *Staphylococcus* relative abundance in nasal samples at onset of irAE. *Staphylococcus* has been implicated as an active regulator of competitive bacterial growth, and was also significantly upregulated in the nares of patients with autoimmune disease [59, 60]; further testing could clarify its role as a surrogate marker of irAE changes. The relative abundance of *Megasphaera* was also found to decrease and increase, respectively, with onset and resolution of toxicities in buccal samples. *Firmicutes* genera, enriched in the baseline gut microbiome of LC patients, has been previously associated with development of CTLA-4 mediated colitis [21, 61]. *Firmicutes*-produced SCFAs have been demonstrated to activate multiple types of G-protein coupled receptors, stimulating either proinflammatory (via MAP kinase) or anti-inflammatory (via β-arrestin 2) responses [14]. Thus, *Megasphaera’s* decrease with onset of toxicity may be indicative of this *Firmicutes* bacteria instigating its own immunologic reaction. Further investigation is needed to confirm.

The patient population of our pilot study contained both NSCLC and SCLC patients. Four SCLC patients were included in different stages of analysis, with three patients who underwent first-line chemoimmunotherapy and one patient with second-line ICI monotherapy; the latter patient progressed on second-line therapy and the three treatment-naïve patients exhibited response. Repeat analysis with removal of SCLC patients slightly decreased significance of but did not fundamentally change our findings. Considering NSCLC and SCLC are cancers of the same organ and carry overlapping environmental risk factors, such as smoking exposure, and that our intent is to identify which members of the respiratory microbiome may be associated with ICI response, we reported results from analysis performed on the aggregate cohort. Given that our findings are similar to those found in microbiome studies of melanoma [19, 22, 48, 62] and renal cell carcinoma [20] patients, it is also reasonable to consider that the microbiome compositions portending response to immunotherapy may also be disease agnostic.

Our study has several limitations that will benefit principally from a larger patient pool. One patient was lost to follow-up shortly after achieving a partial response. Due to follow-up difficulties, several patients were unable to obtain second and third sets of samples for toxicity evaluation. The trends we identified with respect to longitudinal microbiome changes with AEs were drawn from a subset of 5 of 34 possible patients, trends which, albeit interesting, require further validation. Some of the treatment arms, such as LC patients treated with ICI monotherapy, could not be investigated due to small sample size. Based on our current cohort and effect size calculations, we were able to detect 2.16- and 1.23-fold changes in taxa abundance for the ICI response and irAE analyses, respectively [63]. Given that all patients included in ICI response analysis had been treated with chemoimmunotherapy, it is possible that chemotherapy could also have contributed to response rates as well as microbiome alteration. We are currently accruing patients to a subsequent study that will specifically delineate patients treated only with immunotherapy, or are immunotherapy-naïve, for evaluation (ClinicalTrials.gov identifier: NCT04636775, NCT04680377). HC samples used in this analysis also differed from the patient population – controls were relatively younger, though from the same geographic region, and were only able to provide stool samples for comparison. We felt disclosing the LC/HC fecal sample comparison valuable for two specific reasons: first, it is important to confirm that in concordance with other studies the patient population and healthy population have markedly different microbiota at baseline, and second, we feel it important to have two baselines for comparison – a healthy control and a lung cancer baseline control – given that over the course of therapy, many external factors, including chemotherapy, may further perturb that balance. The potential value of such a corroboration, as identified in prior studies [15, 64–66], is that changes in the microbiome may be associated with carcinogenesis as well as with poorer responses to therapy.

Our initial exploratory study may help answer which patients may be at highest risk of developing life-threatening irAEs with use of ICI. More importantly, we hope to identify associations between microbiome constitution and increased immunotherapy efficacy. The potentiation of immunotherapy carries broad implications, including in cancers for which immunotherapy is not currently indicated. A larger cohort to account for confounding factors will further elaborate this: a study focusing on immunotherapy naïve advanced/metastatic NSCLC receiving single agent anti-PD-1/L1 has opened at the University of Kansas Medical Center (due to PI Dr. Jun Zhang’s relocation, ClinicalTrials.gov identifier: NCT04636775). In addition, a separate trial studying the role of the microbiome in predicting toxicity of the anti-PD-L1 agent durvalumab following concurrent chemoradiation in locally advanced NSCLC patients is open to accrual (ClinicalTrials.gov Identifier: NCT04680377; PI Dr. Jun Zhang).

Our research is the first to comprehensively compare oral, nasal and fecal samples among LC patients and report correlations between them with respect to immunotherapy response. It is also notable for being the first to associate *Bifidobacterium* and *Desulfovibrio* with decreased development of irAEs, and further, for being the first to attempt a longitudinal study to evaluate dynamic microbiome changes from various body sites – all these findings will benefit from further investigation in continued preclinical and clinical studies.

## Conclusions

Our study identified multiple promising associations between microbiome alterations and outcomes following ICI therapy in LC patients. Though development of irAEs have been classically associated with treatment response, our findings show that shifts in abundance of specific species may affect only one of these phenomena, potentially decoupling them. Lastly, our study suggests shared correlations between treatment outcomes and disparate microbiome sites in LC patients, which could hold promising predictive value. Our findings warrant further validation with a larger cohort and mechanistic dissection using preclinical models.

## Supplementary Information

**Additional file 1.**

**Additional file 2.**

**Additional file 3.**

**Additional file 4.**

**Additional file 5.**

## Data Availability

Raw data used for analysis has been uploaded to the National Center for Biotechnology Information (NCBI) BioSample database under BioProject ID PRJNA687361, currently pending review and publication. 68 to 93% of input reads remained following denoising with DADA2.

## Abbreviations

AE: Adverse event
CR: Complete response
FDR: False discovery rate
ICI: Immune checkpoint inhibitor
irAE: Immune-related adverse event
HC: Healthy control
LC: Lung cancer
NSCLC: Non-small cell lung cancer
OTU: Operational taxonomic unit
PCoA: Principal coordinate analysis
PLS-DA: Partial least squares discriminant analysis
PR: Partial response
SCFA: Short chain fatty acid
SCLC: Small cell lung cancer
