# Supplementary figures and images for: Prospective correlation between the patient microbiome with response to and development of immune-mediated adverse effects to immunotherapy in lung cancer
Source: BMC Cancer. 2021 Jul 13;21:808. doi: 10.1186/s12885-021-08530-z (PMC8278634; doi:10.1186/s12885-021-08530-z)

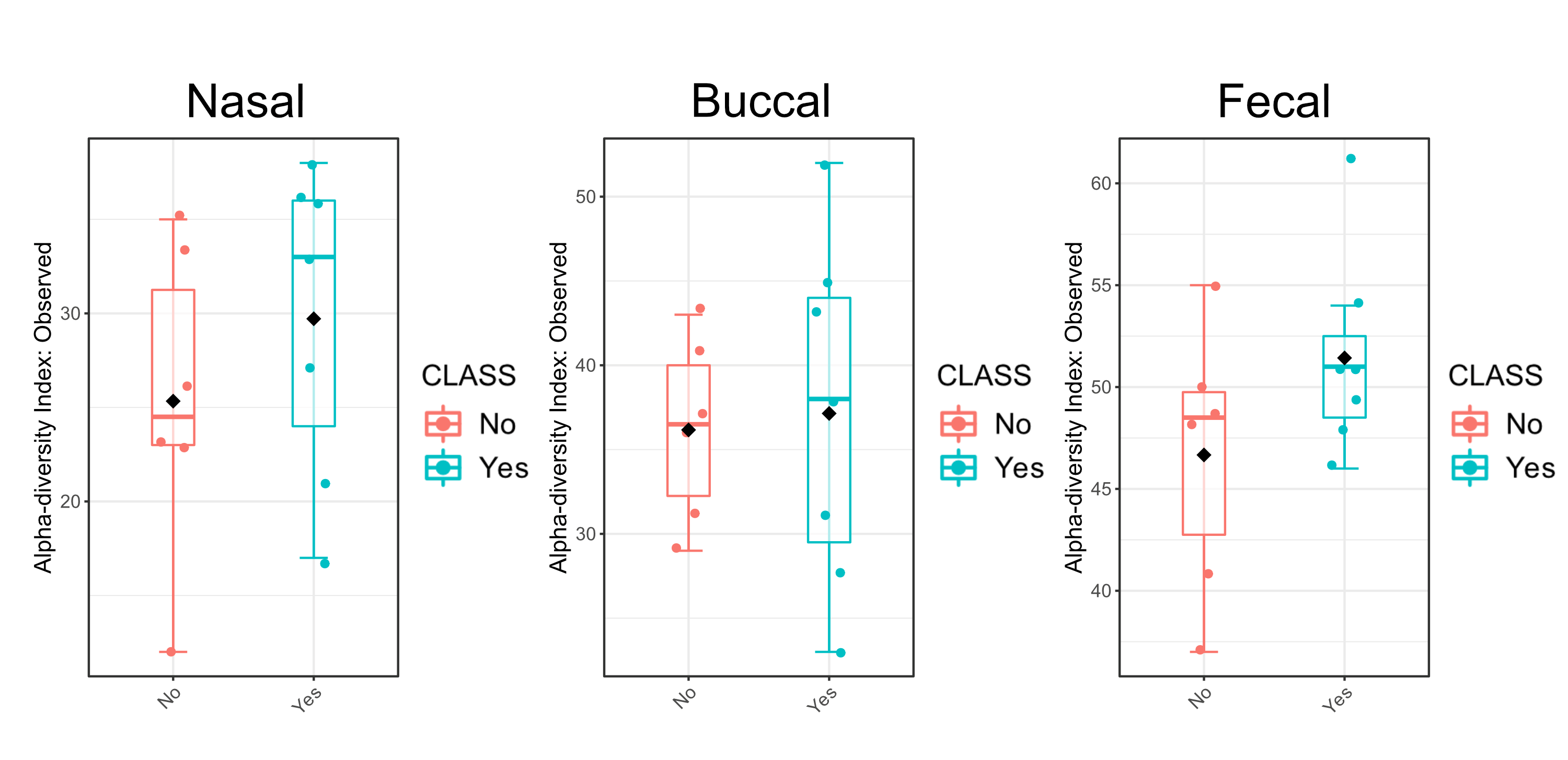

Supplement: Supplementary file 1 — Additional file 1. [file 12885_2021_8530_MOESM1_ESM.tiff]

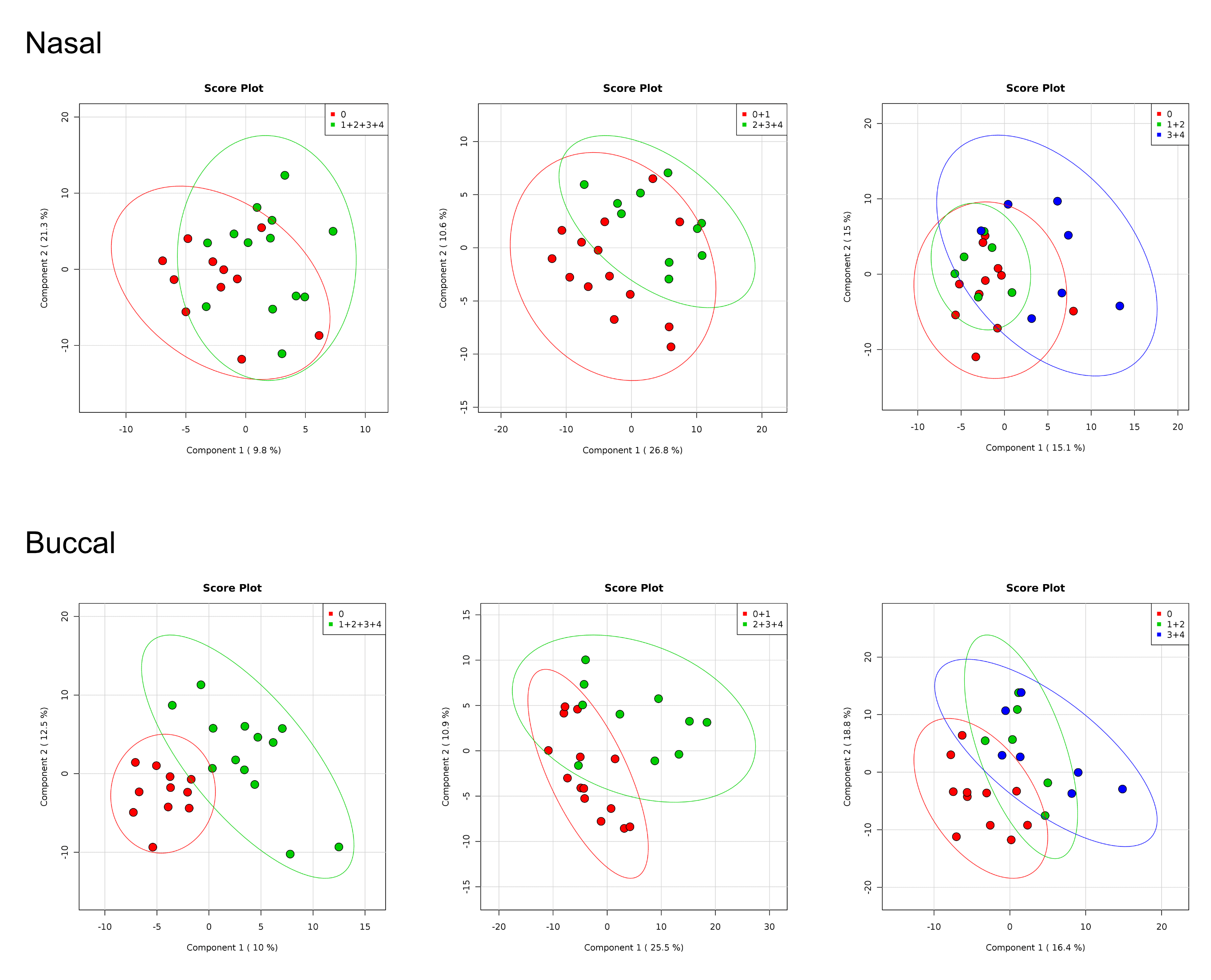

Supplement: Supplementary file 2 — Additional file 2. [file 12885_2021_8530_MOESM2_ESM.tiff]

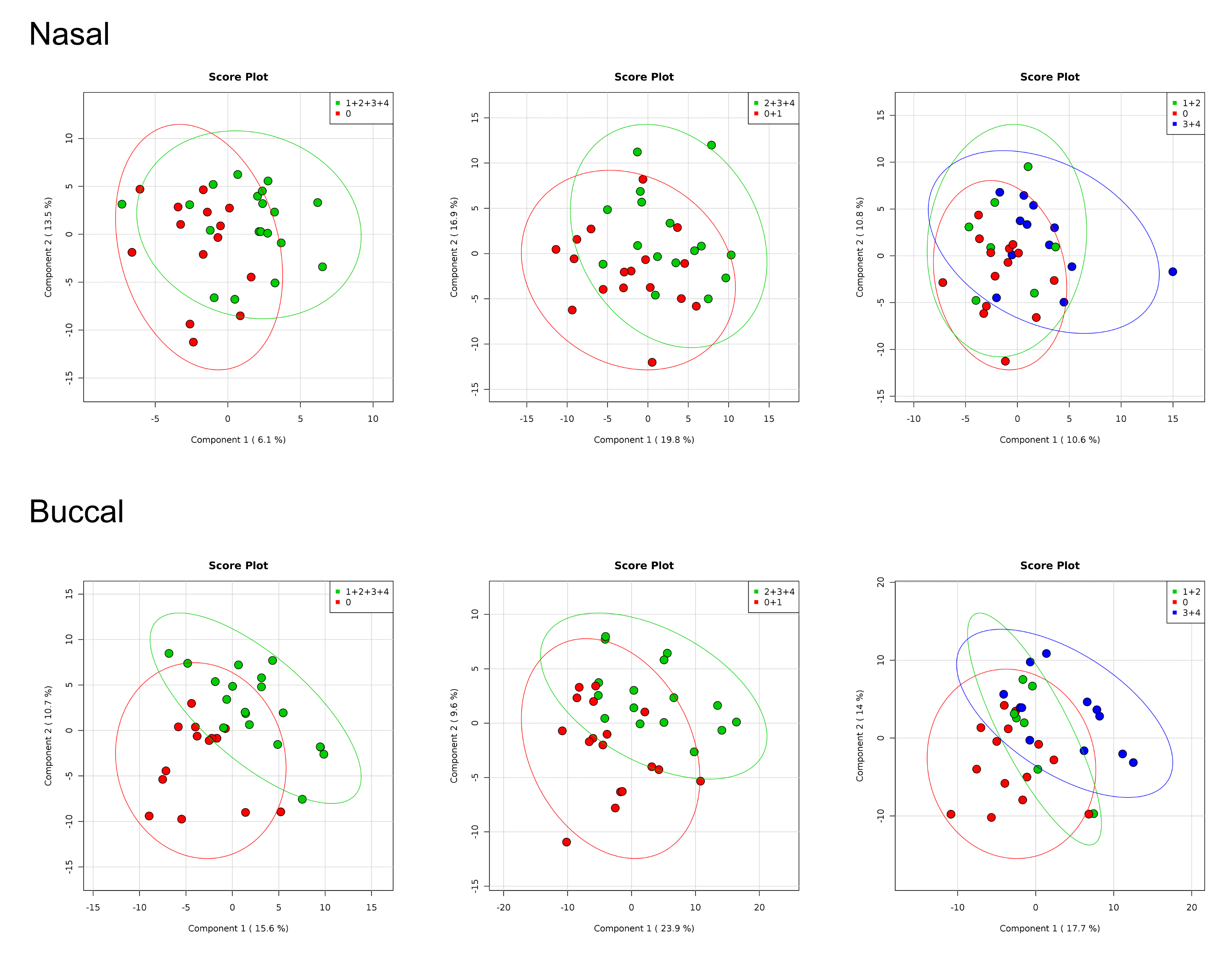

Supplement: Supplementary file 3 — Additional file 3. [file 12885_2021_8530_MOESM3_ESM.tiff]

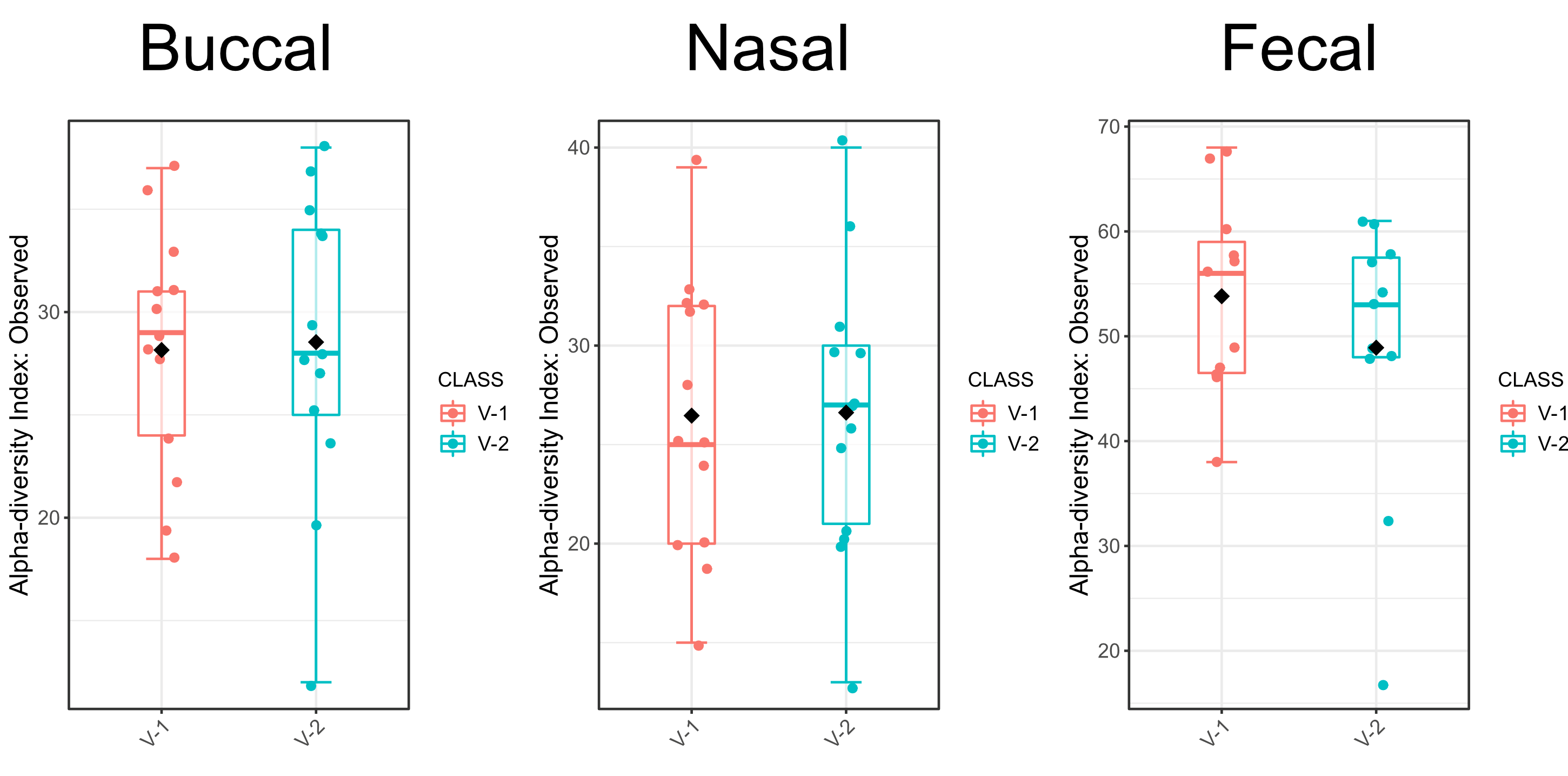

Supplement: Supplementary file 4 — Additional file 4. [file 12885_2021_8530_MOESM4_ESM.tiff]
