# Supplementary material for: Prospective correlation between the patient microbiome with response to and development of immune-mediated adverse effects to immunotherapy in lung cancer
Source: BMC Cancer. 2021 Jul 13;21:808. doi: 10.1186/s12885-021-08530-z (PMC8278634; doi:10.1186/s12885-021-08530-z)
Supplement: Supplementary file 5 — Additional file 5. [file 12885_2021_8530_MOESM5_ESM.pdf]

Supplemental Table 1. Individual Patient and Disease Characteristics.

JZLC.ID	Age at Diagnosis (years)	Sex	Abx within 4 wks of initiation	PPI within 4 wks of start	Probiotic within 4 wks of start	Cancer Type	PD-L1 Status (if tested)	Prior Immunotherapy	Prior Chemotherapy	Cancer Stage at Start of Current Therapy	Line of Current Therapy	Current Immunotherapy	Current Chemotherapy
**JZLC.01**	74	F	N	N	N	Adeno	0	Pembrolizumab	Carboplatin pemetrexed	4A	2	Pembrolizumab	Docetaxel
**JZLC.02**	68	M	N	Y	Y	Squamous	2+, 5% (TC)	No	No	4A	1	Durvalumab	s/p CCRT
**JZLC.03**						**Not enrolled**							
**JZLC.04**	72	F	N	N	N	Squamous	0	No	Carboplatin Etoposide	4A	2	Pembrolizumab	
**JZLC.05**	71	F	N	N	N	Small cell		No	No	Extensive	1	Atezolizumab	Carboplatin Etoposide
**JZLC.06**	75	F	N	N	N	Adeno		No	No	4A	1	Pembrolizumab	Carboplatin Pemetrexed
**JZLC.07**	51	F	N	N	N	Small cell		No	Carboplatin Etoposide	Extensive	2	Pembrolizumab	
**JZLC.08**	72	M	N	N	N	Non small cell, NOS	2+, 10% (TPS)<br>5% (IHC)	No	No	4B	1	Pembrolizumab	Carboplatin Paclitaxel
**JZLC.09**	60	F	N	Y	Y	Adeno	3+, 95% (TC)	Nivolumab	Carboplatin Pemetrexed	4B	3	Pembrolizumab	Docetaxel
**JZLC.10**	86	M	N	N	N	Squamous		No	No	3A	1	Pembrolizumab	Carboplatin Paclitaxel
**JZLC.11**	58	M	N	N	N	Adeno		No	Carboplatin Paclitaxel	2A	2	Durvalumab	s/p CCRT
**JZLC.12**	59	M	Y	N	N	Small cell		No	No	Extensive	1	Atezolizumab	Carboplatin Etoposide
**JZLC.13**						**Not enrolled**							
**JZLC.14**	70	M	N	N	N	Squamous	3+, 5% (TC)<br>Negative (IC)	No	Cisplatin Etoposide	3A	2	Durvalumab	s/p CCRT
**JZLC.15**	63	M	N	N	N	Adeno	3+, 100% (TPS)	No	No	4B	1	Pembrolizumab	Carboplatin Pemetrexed
**JZLC.16**	78	F	N	N	N	Sarcomatoid	2+, 75% (TPS)	No	No	4A	1	Pembrolizumab	Carboplatin Paclitaxel
**JZLC.17**	55	F	N	Y	Y	Small cell		No	No	Extensive	1	Atezolizumab	Carboplatin Etoposide
**JZLC.18**	61	M	Y	Y	Y	Squamous	0	Pembrolizumab	Carboplatin Paclitaxel	4A	2	Pembrolizumab	Gemcitabine
**JZLC.19**	67	F	Y	N	N	Non small cell, NOS	3+, 100% (TPS)	No	No	4B	1	Pembrolizumab	
**JZLC.20**	61	M	N	Y	Y	Squamous	50% (IC)	No	No	3B	1	Durvalumab	s/p CCRT
**JZLC.21**	42	M	N	N	N	Squamous	3+, 75% (TPS)	No	No	4B	1	Pembrolizumab	
**JZLC.22**	72	F	Y	N	N	Squamous	2+, 2-3% (TPS)	No	No	4B	1	Pembrolizumab	Carboplatin Paclitaxel
**JZLC.23**	69	F	N	N	N	Squamous		No	No	2B	1	Durvalumab	s/p CCRT
**JZLC.24**	72	M	Y	N	N	Non small cell, NOS		No	No	3B	1	Durvalumab	s/p CCRT
**JZLC.25**	62	M	N	N	N	Squamous		No	Cisplatin Etoposide	3A	2	Durvalumab	s/p CCRT
**JZLC.26**	49	M	Y	N	N	Large cell	0	No	Carboplatin Paclitaxel	4B	2	Pembrolizumab	Carboplatin Pemetrexed
**JZLC.27**	42	F	N	N	N	Adeno	2+, 60% (TPS)	No	No	4B	1	Pembrolizumab	
**JZLC.28**	47	M	N	N	N	Adeno	2+, 50% (TPS)	No	No	4A	1	Pembrolizumab	
**JZLC.29**	65	M	N	N	N	Adeno	0	Pembrolizumab	Carboplatin Pemetrexed	4B	3	Atezolizumab	Paclitaxel
**JZLC.30**	69	F	Y	N	N	Adeno	0	No	No	3B	1	Pembrolizumab	Carboplatin Pemetrexed
**JZLC.31**	54	F	N	N	N	Squamous and adenocarcinoma		No	Cisplatin Vinorelbine	4A	2	Pembrolizumab	Carboplatin Paclitaxel
**JZLC.32**	74	F	N	N	N	Adeno	2+, 5% (TPS)	No	No	4B	1	Pembrolizumab	Carboplatin Pemetrexed
**JZLC.33**	78	M	N	N	N	Adeno	2+, 20% (TPS)<br>5% (IC)	No	No	4B	1	Pembrolizumab	Carboplatin, pemetrexed
**JZLC.34**	70	M	N	N	N	Squamous	0	No	No	3A	1	Durvalumab	s/p CCRT
**JZLC.35**						**Not enrolled**							
**JZLC.36**	63	F	Y, Z-Pak	N	N	Adeno		No	No	4A	1	Durvalumab	s/p CCRT
**JZLC.37**	78	M	Y, nitrofurantoin	N	N	Squamous and adenocarcinoma	2-3+, 5-10% (TPS)	No	No	4A	1	Pembrolizumab	

s/p CCRT = status-post concurrent chemoradiation therapy.

Supplemental Table 2. Reasons for Sample Collection.

Patient	Sample Set 2	irAE (Grade)	AE (Grade)	Sample Set 3
JZLC-5	Onset of toxicity	Rash (4)	Neutropenia (4)<br>Thrombocytopenia (4)	
JZLC-6	Onset of toxicity	Diarrhea (3)	Neutropenia (3)<br>Fever (3)	Resolution of toxicity
JZLC-8	Onset of toxicity	Nausea/vomiting (3)<br>Diarrhea (3)		
JZLC-10	Onset of toxicity	Myositis (1)<br>Weakness (3)	Neuropathy (3)<br>Thrombocytopenia (2)	Resolution of toxicity
JZLC-11	Onset of toxicity	Rash (3)<br>Pneumonitis (1)		Resolution of toxicity
JZLC-12	Onset of toxicity	Rash (2)	Myalgias (1)	
JZLC-19	Onset of toxicity	Sicca Syndrome (4)<br>Weakness (3)		
JZLC-21	Onset of toxicity	Arthralgias (1)		
JZLC-22	Onset of toxicity	Diarrhea (3)		Resolution of toxicity
JZLC-23	Onset of toxicity	Pneumonitis (3)		
JZLC-24	Onset of toxicity	Pneumonitis (4)		Resolution of toxicity
JZLC-30	Onset of toxicity	Pneumonitis (2)		
JZLC-32	Onset of toxicity	Thrombocytopenia (1)		

irAE = immune-related adverse event.

Note: in the case of JZLC.32, the treating investigator felt the patient's thrombocytopenia could not be clearly attributed to a non-immune etiology. Thus, the patient submitted a second set of samples and treatment with steroids was initiated.

Supplemental Table 3: Collection Omissions

Fecal Sampling

Patient ID	Reason for Omission
JZLC.03	Withdrawn before beginning trial
JZLC.06	Not collected
JZLC.13	Withdrawn before beginning trial
JZLC.17	Received - unable to be sequenced
JZLC.23	Received - unable to be sequenced
JZLC.26	Mislabeled sample, excluded from analysis
JZLC.28	Stool sample was empty
JZLC.33	Stool sample not returned (deceased)
JZLC.34	Received - unable to be sequenced
JZLC.35	Withdrawn before beginning trial

Buccal and Nasal Sampling

Patient ID	Reason for Omission
JZLC.03	Withdrawn before beginning trial
JZLC.13	Withdrawn before beginning trial
JZLC.26	Mislabeled sample, excluded from analysis
JZLC.35	Withdrawn before beginning trial

Supplemental Table 4. Notable differences between healthy controls and lung cancer gut microbiome.

Bacteria	*p*	FDR
UBA1819	4.06E-07	2.41E-05
Eggerthella	6.78E-07	2.41E-05
Ruminococcaceae_UCG_004	7.96E-06	0.00018836
Ruminiclostridium_5	1.47E-05	0.0002609
Eisenbergiella	2.90E-05	0.00041168
DTU089	4.01E-05	0.00047431
Lachnospiraceae_FCS020_group	9.40E-05	0.00095393
Erysipelatoclostridium	0.00016304	0.001447
Lachnospira	0.00020046	0.0015814
Flavonifractor	0.00022482	0.0015962
Barnesiella	0.00026928	0.0017381
Ruminococcaceae_UCG_014	0.0006498	0.0038447
Faecalitalea	0.0010503	0.0057363
Lachnoclostridium	0.001231	0.0062428
Tyzzerella	0.0017741	0.0083973
Erysipelotrichaceae_UCG_003	0.0021165	0.009392
Akkermansia	0.0045814	0.019134
Sutterella	0.0055505	0.021894
Prevotella_9	0.0072068	0.026931
Intestinibacter	0.0099776	0.03542
Butyricimonas	0.012273	0.038691
Slackia	0.012682	0.038691
Holdemanella	0.012892	0.038691
Collinsella	0.013416	0.038691
Escherichia_Shigella	0.013623	0.038691
Lachnospiraceae_NK4A136_group	0.014663	0.039654
Butyricicoccus	0.01508	0.039654
Dialister	0.01629	0.041306
Christensenellaceae_R_7_group	0.021369	0.052316
Ruminococcaceae_UCG_005	0.022431	0.053087
Blautia	0.026031	0.059618
Turicibacter	0.034103	0.075666
Dorea	0.037102	0.079827
Streptococcus	0.039668	0.082835
Coprococcus_1	0.049035	0.099472

FDR = false discovery rate.
